# Epigenetic Studies Point to DNA Replication/Repair Genes as a Basis for the Heritable Nature of Long Term Complications in Diabetes

**DOI:** 10.1155/2016/2860780

**Published:** 2016-02-14

**Authors:** Alexey A. Leontovich, Robert V. Intine, Michael P. Sarras

**Affiliations:** ^1^Division of Biomedical Statistics and Informatics, Mayo Clinic, 200 First Street SW, Rochester, MN 55905, USA; ^2^Department of Biomedical Sciences, Dr. William M. Scholl College of Podiatric Medicine, Rosalind Franklin University of Medicine and Science, 3333 Green Bay Road, North Chicago, IL 60064, USA; ^3^Department of Cell Biology and Anatomy, Chicago Medical School, Rosalind Franklin University of Medicine and Science, 3333 Green Bay Road, North Chicago, IL 60064, USA

## Abstract

Metabolic memory (MM) is defined as the persistence of diabetic (DM) complications even after glycemic control is pharmacologically achieved. Using a zebrafish diabetic model that induces a MM state, we previously reported that, in this model, tissue dysfunction was of a heritable nature based on cell proliferation studies in limb tissue and this correlated with epigenetic DNA methylation changes that paralleled alterations in gene expression. In the current study, control, DM, and MM excised fin tissues were further analyzed by MeDIP sequencing and microarray techniques. Bioinformatics analysis of the data found that genes of the* DNA replication/DNA metabolism process* group (with upregulation of the* apex1, mcm2, mcm4, orc3*,* lig1*, and* dnmt*1 genes) were altered in the DM state and these molecular changes continued into MM. Interestingly, DNA methylation changes could be found as far as 6–13 kb upstream of the transcription start site for these genes suggesting potential higher levels of epigenetic control. In conclusion, DNA methylation changes in members of the* DNA replication/repair process* group best explain the heritable nature of cell proliferation impairment found in the zebrafish DM/MM model. These results are consistent with human diabetic epigenetic studies and provide one explanation for the persistence of long term tissue complications as seen in diabetes.

## 1. Background

Hyperglycemia in patients with diabetes mellitus (DM) (both types 1 and 2) leads to a multitude of complications such as cardiovascular disease, aberrant angiogenesis, retinopathy, nephropathy, neuropathy, and impaired wound healing [1]. Our laboratory has previously reported an adult zebrafish model of type 1 DM that can be used to study the mechanisms underlying the long term complications of the disease. In this model, streptozocin (STZ) induced hyperglycemia (serum  glucose = 315 ± 40.96 mg/dL) is accompanied by the full range of diabetic complications seen in patients with DM [2, 3]. Additionally, we have shown that withdrawal of STZ results in regeneration of pancreatic *β*-cells and the return of previously diabetic fish to a physiologically normal glycemic state within 2 weeks (serum  glucose = 62.5 ± 13.6 mg/dL); however, in contrast, the tissue deficits associated with hyperglycemia persisted permanently (e.g., impairment of angiogenesis [4], impairment of skin wound healing [3], and impairment of limb regeneration [3]). Of relevance to this study, fin regeneration in the DM and MM states has been shown to be significantly impaired and that impairment was found to be due to a decrease in cell proliferation of the fin tissue [2, 3]. Multiple controls indicated that this was a direct effect of *β*-cell necrosis-induced hyperglycemia and not due to any secondary effects of STZ treatment [2, 3]. These findings are consistent with large scale DM clinical trials whose results indicate that once initiated, diabetic complications persist and continue to progress unimpeded even when glycemic control is achieved through pharmaceutical intervention [5–8]. Additionally, this persistence in diabetic tissue deficits has also been supported by multiple lines of experimental laboratory evidence [3, 9–13] and collectively, these data indicate that the initial hyperglycemic period results in permanent abnormalities in the target organs. This harmful phenomenon has been termed metabolic memory (MM) [14, 15] and to date, the underlying molecular mechanism(s) of MM remain unknown.

In the current study, we focused on the molecular mechanisms underlying DM and MM as studied in limb tissue (caudal fin) using our DM/MM zebrafish model because the fin is impaired in its ability to regenerate in the DM and MM states and this impairment was due to a decrease in cell division rates. The objective of the current study was to determine (1) what functional gene groups are prominent during DM that best explain the heritable nature of tissue deficits observed in this model, (2) which genes of these groups persist into MM, and (3) where DNA methylation changes occur in these genes. We found that the zebrafish DM/MM states are associated with changes of the* DNA replication/DNA metabolism process* group. These changes involved (1) upregulation of specific genes of this group (*apex1, dnmt1, mcm2, mcm4, orc3*,* pola2*, and* lig1*) in the DM and MM states and (2) alterations in the DNA methylation patterns of all of these genes. Differentially methylated DNA regions (MRs) were found as far as 6–13 kb upstream of the transcription start site for the affected genes but were also found within the gene proper in one case.

The overriding hypothesis of these studies was that “*persistent tissue changes in the DM/MM states correlate with hyperglycemia-induced DNA methylation changes that are associated with specific functional gene groups related to the heritable nature of MM*.” To test this hypothesis we employed (1) MeDIP sequence analysis in combination with concomitant gene expression analysis, (2)* in silico* determination of prominent functional groups of differentially expressed genes observed in the DM and MM states, and (3) further bioinformatics analysis of methylated genomic regions upstream and downstream of the transcription start site of those genes that had been identified. This approach has allowed us to gain insight into the underlying epigenetic mechanisms that might explain the persistence of impaired tissue function(s) that occur in the DM state and continue into the MM state using the zebrafish diabetic model. The study focused on the zebrafish caudal fin because previous studies have established that fin tissue is best suited for experimental creation of a “pure” metabolic memory tissue [2, 3]. Other tissues of the zebrafish (e.g., kidney, retina, and skin) enter the MM state following *β*-cell regeneration, but unlike the fin; with these it is more difficult to form a tissue that lacks the residual molecules that were created in the original hyperglycemic state such as ROS (Reactive Oxygen Species) and AGEs (Advanced Glycation Endproducts). The presence of such pathology-inducing molecules as ROS and AGEs introduces complicating variables that makes evaluation of the epigenetic effects more difficult to interpret.

## 2. Methods

### 2.1. Zebrafish Husbandry, STZ Injection, and Fasting Blood Glucose Determination

The maintenance of zebrafish stocks (*Danio rerio*), the induction of hyperglycemia, blood glucose determinations, and fin regeneration methodology were performed as previously described [2]. For intraperitoneal injection an insulin syringe with a 28.5-gauge needle was used to deliver 0.3% streptozocin (STZ) (Sigma, S0130) solution in 5 mM citrate buffer, pH 5.0 to a dose of 350 mg/kg (70–150 microliters dependent on weight). Control fish were injected with a like volume of citrate buffer. The fish used in these studies were approximately 4–7 months of age. Fasting blood glucose level parameters used included the following: normal, 60 mg/dL; DM, 315 mg/dL; and MM, a return to 60 mg/dL. All procedures were performed following the guidelines described in “Principles of Laboratory Animal Care” (NIH publication number 85-23, revised 1985) and approved IACUC animal protocols 08-19 and B11-16 for these studies.

### 2.2. Creation of a “Pure” Metabolic Memory Tissue for Molecular Analysis

**Figure 1:**
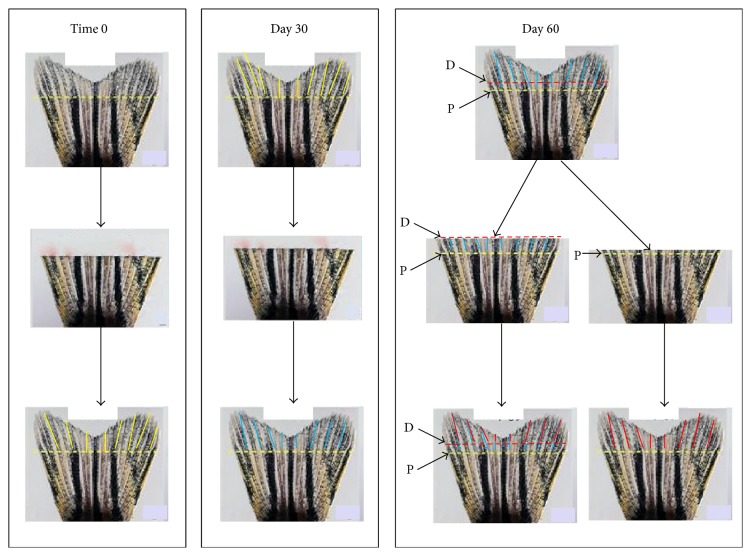
Schematic of amputation scheme employed to generate metabolic memory fin tissue. At Time 0, caudal fins were amputated and allowed 30 days for regrowth (Time 0 panel). At 30 days, the fins were again amputated and allowed an additional 30-day period of regenerative growth (Day 30 panel). This tissue is called metabolic memory tissue as it was generated outside of the hyperglycemic state. At 60 days, the groups were split into two fin sets and were cut either proximally (yellow dashed line, P) or distally (red dashed line, D) [Day 60 panel]. When analyzed for residual ROS and AGE molecules, the growth from the proximal and distal cuts (bottom left side of Day 60 panel, indicated by blue and red bars) had no residual ROS or AGE molecules, while tissue of the original fin (inferior to the proximal cut) did have residual ROS and AGE molecules [2, 3]. Excision of the distal cut growth (red bar area) was used for all DNA methylation sequence analysis and gene expression (microarray) analysis because it was pure metabolic memory region tissue (with no residual molecules from the original hyperglycemic state) and was clearly morphologically distinct from the original hyperglycemic fin tissue (each cut line can be observed in the regenerate fin tissue).

As described in previous studies [2, 3], we have designed a way to obtain MM tissue that lacks any residual molecules that are created in the original DM state such as ROS and AGEs (common to diabetes in all vertebrate species [16]). As shown in Figure 1, this involves repeated amputation and regeneration of fin tissue that has entered the MM state following regeneration of *β*-cells. As stated, analysis of this MM fin tissue finds that it lacks any of the residual molecules found generated in the original DM state so that this regenerated fin tissue is in a “pure” MM state without the complicating signals existing in the original hyperglycemic DM tissue [2, 3].

### 2.3. RNA Extraction

RNA extraction procedures followed that reported previously by our laboratory without exception [2, 3, 17]. Triplicate samples of 15 caudal fin samples were obtained from intact fin tissue for (1) acute diabetic fish (DM), (2) 60-day metabolic memory fish (MM), and (3) controls (CTRL). For clarification purposes, (1) CTRL fish represents normal adult zebrafish vehicle injected for the diabetic group, (2) DM represents zebrafish that have been induced into an acute diabetic state (for a three-week period) using STZ, and (4) MM represents zebrafish that were initiated and were maintained in a diabetic state for the three-week period but returned to a euglycemic state following withdrawal of STZ (referred to as MM fish throughout the paper). At 30 days after drug removal, the caudal fins were amputated to eliminate any residual molecules within the fin tissue from the original hyperglycemic period as previously described [2, 3]. After an additional 30-day growth phase, the fish were considered to be the final MM group to be used for various analyses of the excised “pure MM” fin tissue (see Figure 1).

### 2.4. Microarray Analysis

Extracted RNA (procedure described above) was used to probe the previously established Affymetrix GeneChip® Zebrafish Genome Array which contains 15,509 probe sets designed to interrogate expression of 14,900* Danio rerio* transcripts [2, 3, 17]. Microarray analysis was conducted according to manufacturer's instructions for the Affymetrix 3′ IVT Express Kit and all subsequent procedures were followed as previously reported by our laboratory [2, 3, 17] with the modification that (1) we first determined all genes with altered expression in the DM state (as compared to controls) and then from this group (2) we determined which of these genes maintained altered expression in the MM state. In our previous studies, this analysis was performed simultaneously for the DM and MM states [2, 3, 17]. This new method was utilized because it was more inclusive of all genes initially affected by hyperglycemia.

### 2.5. DNA Isolation and Methylated gDNA Sequencing

Triplicate samples of 15 caudal fins were obtained from control, DM, and MM zebrafish caudal fin tissue (conditions for control, DM, and MM were the same as described for Section 2.3) and immediately processed via the PureLink Genomic DNA Mini-Kit (Life Technologies). Methylation DNA immunoprecipitation sequencing (MeDIP) and initial sequence analysis was performed as previously described by our laboratory [2, 3, 17].

### 2.6. Gene Enrichment Analysis from Zebrafish Microarray Analysis

Gene function enrichment analysis was performed using DAVID Bioinformatics Resources 6.7 [18]. Additionally, the STRING 9.1 online bioinformatics resource was also utilized to visualize the results of this analysis, namely, representing relationships between specific genes and the significance of their interaction as described by Franceschini et al. [19].

### 2.7. Methylation Analysis of Zebrafish gDNA from Control, DM, and MM Groups

Analysis of FASTQ files generated by Illumina Genome Analyzer IIx was performed using Galaxy (https://usegalaxy.org/) as published by Goecks et al. [20] and Blankenberg et al. [21]. The MACS algorithm [22] of the Galaxy program was specifically applied for analysis of DNA methylation changes among the groups studied.

The conditions for application of the algorithm were set with the following parameters: effective genome size: 1,480,000,000 bp; tag size: 25 or 32 depending on the results of quality control and trimming; band width: 300; *P* value cut-off for peak detection: 1*e* − 05; MFOLD: 30; regions around the peak region to calculate maximum lambdas local lambda: 1000, 5000, and 10000; mapping of methylated regions to zebrafish genome and visualization of the results were performed using UCSC genome browser and IGV genome browser as described by Thorvaldsdóttir et al. [23] and Robinson et al. [24].

## 3. Results

### 3.1. Genes of the DNA Replication and DNA Metabolism Functional Categories That Are Differentially Expressed in the DM State as Compared to Controls

Gene enrichment analysis was performed to determine the functional categories that were enriched by the genes that were identified in the microarray studies. Using a cut-off of 2-fold differential expression in the DM condition relative to control with a false discovery rate (FDR) cut-off of 0.05, we identified a number of functional groups (see Table 1). The first four groups were related to (1)* protein folding and binding*, (2)* RNA translation*, (3)* protein transport and localization*, and (4)* cell homeostasis*, while the next functional category was related to (5) the* DNA replication/DNA metabolism process* group. We focused further analysis on the latter group (enrichment score of 2.7, Table 1) because of its important relationship to understanding the mechanisms underlying the heritable nature of MM in the zebrafish. While the other groups would have a role in MM, they cannot explain the heritable basis of the cell proliferation deficits in MM.

We identified 51 genes as being overrepresented in the functional category* DNA replication/DNA metabolism process* group (Supplemental Table 1 in Supplementary Material available online at http://dx.doi.org/10.1155/2016/2860780). Again, a parameter of at least 2-fold differential expression in the DM condition relative to controls with a FDR cut-off of 0.05 was used for gene selection.

**Figure 2:**
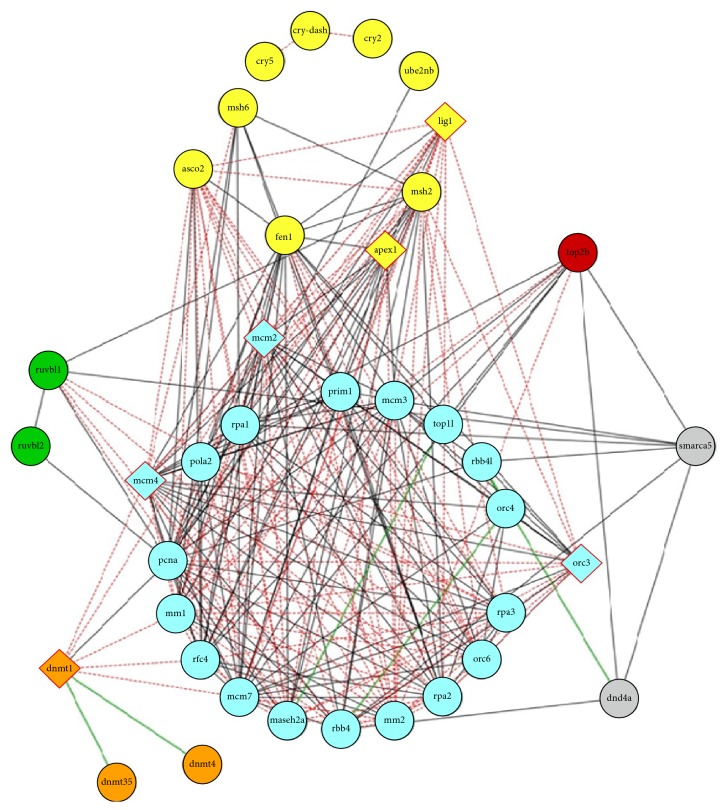
Gene network showing associations between genes that were differentially expressed in the DM and MM states relative to controls (genes of Figure 2 are listed in Supplemental Table 1). Nodes of the network represent genes that are grouped by their involvement in biological process as defined by the Gene Ontology and are colored accordingly (light blue denotes genes involved in DNA replication; gray, in chromatin remodeling; orange, in DNA methylation; green, in DNA recombination; yellow, in DNA repair; and brown, in DNA topological change). Diamond-shaped nodes represent genes with differentially methylated regions. Gene interactions confirmed experimentally and by coexpression are shown as black lines connecting nodes; red dotted lines show associations derived only from coexpression data; and green lines denote interactions confirmed by only experimental evidences.

We next determined that genes overrepresented in the functional category* DNA replication/DNA metabolism process* group do, in fact, have associations with each other based on analysis using the STRING 9.1 program that determines gene associations based on published human and mouse research literature [19]. The diagram shown in Figure 2 was generated by the STRING 9.1 program and depicts the interrelationship of all genes listed in Supplemental Table 1. It is to be noted that, among the 51 genes reported to interact in this group, only a subset were found to be upregulated in both the DM and MM states of our study as depicted by the diamonds in Figure 2 (discussed in more detail in Section 3.2).

### 3.2. Identification of Genes Differentially Expressed in the DM State That Remained Differentially Expressed in the MM State

**Figure 3:**
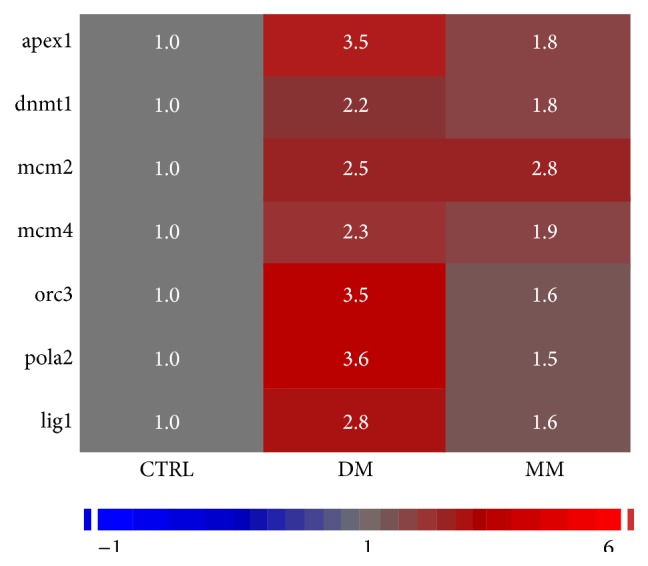
Heat map of expression of genes that are with altered expression in both the DM and MM condition and that are critical members of the* DNA replication/DNA metabolic* process group. As indicated, all genes listed were found to be upregulated.

Of the functionally related genes of Figure 2, we selected those genes that had altered expression in the DM state and then maintained this altered expression in the MM state (relative to controls) using a cut-off of 1.5-fold differential expression in the MM state condition with a FDR cut-off 0.05. A 1.5-fold differential expression cut-off was used for the genes affected in the MM state because it was consistent with the criteria applied to the analysis of the MM state in our previous published studies [2, 3, 17]. Additionally, this approach was chosen with the logic that MM would encompass genes whose altered expression in the DM state would abnormally persist in the MM state [2, 3, 17]. Six genes met this cut-off and are depicted in the heat map shown in Figure 3. These genes included* apex1*,* dnmt1, mcm2, mcm4, orc3*,* pola2*, and* lig1*. All of these genes were found to be upregulated. These genes can further be classified into two functional groups to include (1) DNA replication and/or repair (*apex1, mcm2, mcm4, orc3*,* pola2*, and* lig1*) and (2) DNA methylation (*dnmt1*, a methyltransferase that functions during the replication process) based on the criteria of the applied algorithms.

### 3.3. Methylation Analysis of the Zebrafish Genes Found to Have Altered Gene Expression in the DM and MM States

**Figure 4:**
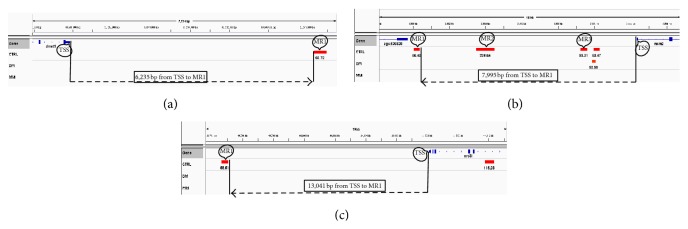
Methylated DNA regions (MRs) of zebrafish gene loci for* dnmt1* (a),* mcm2* (b), and* orc3* (c). Panels (a), (b), and (c) were prepared with IGV genome viewer. The scale on the top shows chromosomal coordinates. This program schematically represents the structure of the gene using conventional elements: (1) blue line represents introns and (2) blue blocks on the line represent exons. Circles with the “TSS” point to the transcription start site and the dashed arrow points in the direction of 5′ to 3′ of the DNA strand with the arrow pointing to the MR farthest upstream of the TSS as indicated for each diagram (bp distance indicated in the box above the dashed arrow). Three tracks separated by gray lines below gene track show localization of MRs for each of the three samples to include control (CTRL), diabetic state (DM), and metabolic memory state (MM). Methylated regions detected by the MACS algorithm are shown as red boxes. Those upstream of the TSS that are lost in both the DM and MM states are numbered (MR1, MR2, etc.) and shown within circles over the red bars. The sequence counts for MACS peaks are shown under each MR. (a)* dnmt1*, (b)* mcm2*, and (c)* orc3*. (a)* dnmt1*, chromosomal coordinates are chr3:53,494,972-53,510,920; (b)* mcm2*, chromosomal coordinates are chr22:3,992,357-4,013,603; and (c)* orc3*, chromosomal coordinates are chr17:44,806,126-44,836,951.

All members of the* DNA replication/DNA metabolism process* group found to have altered expression (upregulated) in the DM state and persisted in having altered expression (upregulated) in the MM state (Figure 3) were then analyzed as to the differential DNA methylation patterns observed between the control, DM, and MM states. Methylated DNA regions are abbreviated as “MRs” in this paper. These genes were specifically analyzed in regard to (1) the position of MRs, (2) the percent GC content, and (3) the location of CpG islands relative to overall methylated content. In regard to the first point, our analysis found that MRs could be found (1) upstream of the transcription start site (TSS), (2) within the gene proper, and/or (3) downstream of the termination codon. The MRs for* dnmt1, mcm2*, and* orc3* are shown in Figure 4 and are depicted as red bars in that figure for the control, DM, and MM states.

**Figure 5:**
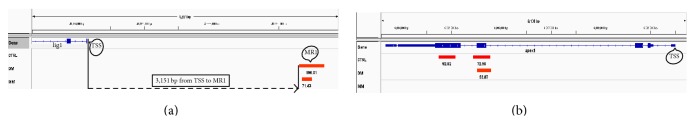
Methylated DNA regions (MRs) of zebrafish gene loci for* lig1* (a) and* apex1* (b). Panels (a) and (b) were prepared with IGV genome viewer. The scale on the top shows chromosomal coordinates. This program schematically represents the structure of the gene using conventional elements: (1) blue line represents introns and (2) blue blocks on the line represent exons. Circles with the “TSS” point to the transcription start site and the dashed arrow points in the direction of 5′ to 3′ of the DNA strand with the arrow pointing to the MR farthest upstream of the TSS as indicated for each diagram (bp distance indicated in the box above the dashed arrow). Three tracks separated by gray lines below gene track show localization of MRs for each of the three samples to include control (CTRL), diabetic state (DM), and metabolic memory state (MM). Methylated regions detected by the MACS algorithm are shown as red boxes. Those upstream of the TSS that have a loss of methylation in the DM and/or MM states are numbered (MR1, MR2, etc.) with the MACS peak ID numbers under each MR. (a)* lig1*, (b)* apex1*. The MRs of* apex1* are all located within the ORF and are not numbered, although they show a loss of methylation in the DM and/or MM as seen with* dnmt1*,* mcm2*, and* orc3*. In contrast* lig1* which did show upregulation in gene expression in the DM and MM states showed the opposite methylation pattern with the control state showing no MRs while the DM and MM states showed the appearance of MRs (Figure 5(a)).

**Figure 6:**
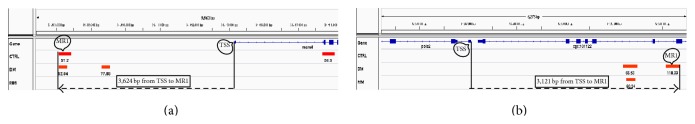
Methylated DNA regions (MRs) of zebrafish gene loci for* mcm4* (a) and* pola2* (b). Panels (a) and (b) were prepared with IGV genome viewer. The scale on the top shows chromosomal coordinates. This program schematically represents the structure of the gene using conventional elements: (1) blue line represents introns and (2) blue blocks on the line represent exons. Circles with the “TSS” point to the transcription start site and the dashed arrow points in the direction of 5′ to 3′ of the DNA strand with the arrow pointing to the MR farthest upstream of the TSS as indicated for each diagram (bp distance indicated in the box above the dashed arrow). Three tracks separated by gray lines below gene track show localization of MRs for each of the three samples to include control (CTRL), diabetic state (DM), and metabolic memory state (MM). Methylated regions detected by the MACS algorithm are shown as red boxes. MRs upstream of the TSS that have a loss of methylation in the DM and/or MM states are numbered (MR1, MR2, etc.) with the MACS peak ID numbers under each MR. (a)* mcm4*, (b)* pola2*. As shown in Figure 6(b),* pola2* mimicked* lig1*. Like* lig1*,* pola2* displayed upregulation of gene expression in the DM and MM states but showed no differential methylation pattern in the control state. Also like* lig1*,* pola2* was seen to have MRs detected in the DM and/or MM states.

In general, the trend for* dnmt1*,* mcm2,* and* orc3* was for MRs upstream of the TSS to be methylated in the normal state but to have a reduced methylated CpG content in the DM and MM states (MRs that are methylated in the normal state and have a reduced methylated CpG content in both the DM and MM states are indicated by circles pointing to the MR with the gene's name listed under the gene proper (Figure 4)). We did find some exceptions to this trend for MRs within the gene proper. In the case of* dnmt1* (see Figure 4(a)), one methylated MR was found in normal tissue that was upstream of the TSS and this MR had a reduced methylated CpG content in the DM and MM states. For* mcm2* (Figure 4(b)), four MRs were identified and all four were upstream of the TSS. Three of the MRs upstream of the TSS were methylated in normal tissue but had a reduced methylated CpG content in both the DM and MM states, while the fourth upstream MR remained methylated in DM but had a reduced methylated CpG content methylated in MM. In the case of* orc3* (Figure 4(c)), one MR was found upstream of the TSS while another was within the gene proper. Both of these MRs were methylated in the normal state but had a reduced methylated CpG content in both the DM and MM states. MRs for* lig1, apex1, mcm4*, and* pola1* are shown in Figures 5 and 6.

The MRs for* lig1* and* apex1* are shown in Figure 5 and the MRs for* mcm4* and* pola1* are shown in Figure 6. The MRs for* mcm4* (Figure 6(a)) followed the trends shown for* dnmt1, mcm2*, and* orc3*. While each of these genes showed upregulation of their mRNA expression, there were variations in their methylation patterns as compared to* dnmt1, mcm2,* and* orc3*. In the case of apex1, there was a loss of MRs in the DM and/or MM states, but this occurred in the ORF and not upstream of the TSS. In contrast,* lig1* and* pola2* showed opposite DNA methylation patterns to that of the other genes. In their cases,* lig1* and* pola2* had no MRs in the control state while MRs appeared in the DM and/or MM states, indicating a hyperglycemia-induced hypermethylation which persisted into the MM state for* pola2*.

**Figure 7:**
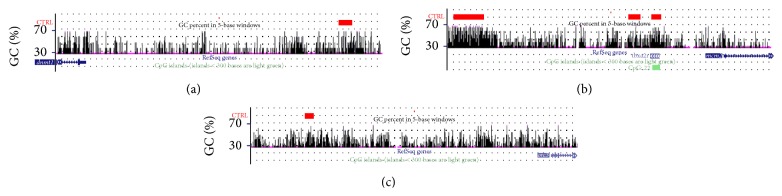
DNA methylation patterns within MRs of genomic areas upstream of the TSS for the* dnmt1*,* mcm2*, and* orc3* genes (Panels (a), (b), and (c), resp.). The zebrafish genes (and associated upstream genomic regions) were analyzed in regard to (1) methylated regions, (2) percent GC content, and (3) CpG islands. CpG islands are defined as containing at least 50% of CG dinucleotides within a region of at least 200 bp. As in Figure 4, red bars represent methylated regions (MRs) as defined by the MACS algorithm for the zebrafish genes and black vertical bars represent GC content in the selected genomic region. For* mcm2*, the three red bars represent MRs 1, 2, and 3 as depicted in Figure 4. Green bars (when present) indicate CpG islands (CpG island is a region with at least 200 bp and a GC percentage that is greater than 50% and with an observed-to-expected CpG ratio that is greater than 60%). Notice that the red bars overlay areas of elevated GC content that are not classified as canonical CpG islands except for the far right red bar for* mcm2*, as indicated by the lower green bar to the right of center in Panel 7(b).

We then focused on representative members of the DNA replication/repair (*mcm2* and* orc3*) and DNA methylation (*dnmt1*) groups to conduct more detailed analysis of CpG methylation patterns within MRs (see Figure 7). This analysis involved application of the UCSC genome browser to identify canonical CpG islands (length > 200 bp, GC content > 50%, and Obs_CpG_/Exp_CpG_ > 0.60) [25] and use of the EMBOSS software to find regions shorter than 200 bp with observed/expected CpG abundance ratio above 0.6 [25, 26]. This second type of less than 200 bp is more easily seen in Figure 7(b) for zebrafish* mcm2* (CpG island region indicated by the green bar in the figure). Many high CpG content regions associated with the zebrafish genes we studied could not be classified as being either canonical or of the second type, but these regions could be found to be methylated based on the zebrafish MeDIP sequencing data.

## 4. Discussion

This study was designed to investigate the underlying mechanisms that could explain the heritable nature of diabetic metabolic memory. To this aim, gene enrichment analysis found a number of functional gene categories arising from our gene expression analysis of the control and DM fish. Of these functional gene categories we focused on the* DNA replication/DNA metabolism process* group because of its clear importance to the heritable nature of MM. While the other groups clearly contribute to the tissue dysfunctions observed in the DM and MM states, they do not explain the heritable basis of the pathology. When this group was analyzed in terms of transcripts that had an altered expression pattern in the DM group versus the control group, we found the genes listed in Supplemental Table 1. The validity of this list was enhanced when we determined, using gene network analysis, that these genes are interrelated based on current mouse and human literature. Of these genes, we found a subset whose altered expression pattern persisted into the MM state. These transcripts included genes related to (1) DNA replication/repair (*apex1, mcm2, mcm4, orc3,* and* pola2*) and (2) DNA methylation (*dnmt1*, a methyltransferase that functions during the replication process). We found that these genes showed a transcript upregulation pattern and all were found to have an altered methylation pattern associated with their loci (either upstream of the TSS or within the gene proper). As an extension of our previously published MeDIP global DNA methylation sequencing [3], we found that each of the genes with altered methylation patterns in the control, DM, and MM states was methylated upstream of its TSS in the control state but typically had a selective reduction of methylated CpGs in the DM state and this reduction in methylated CpGs was maintained in the MM state. The exception to this trend was* lig1* and* pola2* which both showed an increase in DNA methylation in the DM and MM states as compared to controls. This follows trends reported in the literature for DNA methylation changes in disease states, with hypomethylation being predominate over hypermethylation [27]. While any changes in DNA methylation patterns lead to an alteration in gene expression that subsequently leads to tissue dysfunctions [3, 17, 27], it is important to note that while increased methylation often results in decreased expression of the gene, this result is not always the case [28]. As indicated, this was the case for* lig1* and* pola2* in which an increase in gene expression was found in combination with increased methylation for the DM and/or MM states.

We also analyzed representative members of these two functional groups (*dnmt1, mcm2*, and* orc3*, as shown in Figure 7) in more detail to determine the specific CpG methylation patterns associated within the gene's loci. The reduction in DNA methylation of* dnmt1, mcm2*, and* orc3* occurred in CpG rich regions and/or CpG islands depending on the gene analyzed. In the case of zebrafish* mcm2* that contains multiple methylated regions (MRs), some of these MRs overlapped with CpG islands, while, in the case of* dnmt1* and* orc3*, CpG islands were not identified in MRs because the number of the CpG dinucleotide clusters was less than 200 in the regions upstream of the TSS (200 GC dinucleotides being the cut-off for a CpG island based on the criteria: length > 200 bp, GC content > 50%, and Obs_CpG_/Exp_CpG_ > 0.60) [25]. This finding raises questions as to the actual relationship between CpG rich areas (those defined as CpG islands and those not defined as CpG islands) and the methylation process as pertaining to regulatory mechanisms affecting gene expression. It is interesting to note that methylation of CpG rich areas and CpG islands was restricted to a limited number of regions within the genomic area upstream of the TSS and was not seen in all areas that could have been methylated based on CpG content. How hyperglycemia induces this specific type of methylation is unknown but could be related to chromatin 3-dimensional structure. In this context, some CpG rich areas may be more accessible to the methylation machinery than other CpG rich areas in the same gene due to regional chromatin structural differences [29]. Moreover, recent studies related to histone-methylation indicate that higher levels of genomic control (termed, epigenetic control regions (ECRs) [30, 31]) exist for regulation of epigenetic processes [30, 31]. Whether such ECRs have a role in the epigenetic processes described in our studies remains to be determined.

As indicated in our previous 2012 article [3], not all genes in the zebrafish DM/MM model have methylation changes following hyperglycemia. Hyperglycemia is loci specific in terms of which genes are targeted for methylation changes. Does this mean that only genes with differential methylation patterns are susceptible to dysregulation of their expression? In this regard, it is important to note that network analysis indicates that genes of the DNA replication/repair group are all functionally interrelated. This means that changes in the methylation pattern of one particular gene would change that gene's expression and this changed gene expression pattern could in turn affect other genes that had no methylation changes associated with them because all genes in the network are functionally interconnected. Therefore a gene does not need to have an altered DNA methylation pattern for sustained upregulation or downregulation of its expression in the MM state. Evidence for this condition comes from the studies of Dehde et al. [32] who found that* pola2* has direct interactions with* mcm2* during DNA replication and therefore methylation changes in one can affect the other gene regardless of whether both genes have methylation changes. Compensatory expression mechanisms could trigger gene expression pattern changes in such a case. The regulatory effects of DNA methylation as related to changes in gene expression patterns are poorly understood and require additional study.

As an extension of the above discussion, it is important to note that many of the MRs we have identified in the DNA replication and repair genes are far upstream of each gene's transcription start sites. The functional importance of these MRs in the regulation of the genes we have identified has not been established in our studies and therefore, our data are correlative in this regard. Therefore, as an important next step in our studies, we will be applying molecular techniques that allow us to prevent MRs from being methylated* in vivo* so that we can then determine how this modification affects expression of the gene of interest. Prevention of methylation of the MR coupled with subsequent gene expression analysis (as compared to gene expression observed in the unmodified DM/MM state) will allow us to functionally tie MRs to specific genes. This approach will be the foundation for future studies using the zebrafish DM/MM model as well as accessible human tissues obtained from patients with diabetes (e.g., peripheral blood cells, exempt surgically disposed tissue).

The data obtained from the zebrafish model points the way to potential mechanisms in the human diabetic condition. In this regard and given the fact that, in comparison to the human genome, approximately 70% of human genes have at least one obvious zebrafish orthologue [33], the bioinformatics analysis applied to the zebrafish diabetic model may provide insight into the human disease state. This is also reinforced by the fact that zebrafish glucose regulation mimics that of all mammals to include the human. Moreover, reductions in DNA methylation regions can also be found in the genome of human diabetic patients [34]. In this particular case, hypomethylation in the promoter region of the Connective Tissue Growth Factor (CTGF) gene has been reported for patients with T2 DM [34]. CTGF is a known regulator of cell proliferation as related to the process of angiogenesis and therefore is functionally tied to DNA replication and repair mechanisms.

In regard to the human diabetic state, it is well known that long term complications related to alterations in cell proliferation rates are characteristic of both type 1 and type 2 diabetic patients [35–40] and our current findings provide a molecular basis to help explain this type of tissue dysfunction due to changes in the transcription of genes fundamental to cell division. It should be noted that cell proliferation rate alterations as is seen in angiogenesis have been reported to include both increased and decreased cell division rates in both type 1 and type 2 DM, depending on the tissue studies [41]. Changes in cell proliferation rates have been highlighted in the zebrafish DM/MM model and these altered rates have been reported to affect a number of hyperglycemia-induced dysfunctional tissues of the model to include the CV system [4], visual system [2], and limb tissues [2, 3]. The data of the current study in the zebrafish DM/MM model indicate that future studies in human diabetic tissues should focus on DNA methylation changes in (1) genes that are susceptible to methylation changes and (2) genes that regulate cell proliferation to establish a potential basis for such deficits associated with the long term compilations seen in both type 1 and type 2 diabetic patients.

As a final note, it must be remembered that secondary complications in diabetes arise from a multitude of cellular, biochemical, and molecular factors as discussed by Brownlee over a decade ago [16] and more recently by Fowler [1] who focused on complications related specifically to the cardiovascular system. Even in the case of epigenetic mechanisms, DNA methylation is just one process among many (e.g., histone modifications and microRNA changes). Consequently, it is most likely that clinical approaches to the treatment and prevention of the secondary complications seen in diabetes will require multifaceted therapies, with strategies targeting DNA methylation changes being just one of many.

## 5. Conclusions

In conclusion, these studies provide a molecular basis for the many studies reporting altered cell proliferation rates in the long term diabetic condition. They point to DNA methylation changes and concomitant gene expression changes being tied to problems in DNA replication/repair genes, which are consistent with the alterations in cell proliferation observed in both the DM and MM states of the zebrafish DM/MM model. These observations may extend to the human diabetes conditions, where alterations in such genes as MCM2 have also been observed to occur [42]. Future studies will expand on these findings by translating that found in the zebrafish DM/MM genes to human genes whose DNA methylation patterns are altered in the diabetic state (such as those found with the human CTGF gene of patients with T2 diabetes). The ultimate aim will be to (1) determine that methylation changes in MRs are functionally tied to the genes of interest and (2) determine the effect of these changes on the ability of transcription factors to bind to their DNA binding sites. The proposed underlying mechanism in this latter case relates to transcription factor binding dysregulation via causing alterations in gene expression. These impairments in transcription factor binding would then lead to tissue dysfunction as observed in the long term disease.

## Supplementary Material

Supplemental Table 1: The supplemental table 1 data provides the names of the 51 genes that were identified as being over-expressed in the functional category *DNA replication/DNA metabolism process group*. A selection criteria of at least 2-fold differential expression in the DM condition relative to controls with a FDR cut-off of 0.05 as used. The table lists for each column, the ENTREZ ID #, the Gene Symbol, and the Gene Name. Some genes are unknown (indicated by *zgc*).

## Figures and Tables

**Table 1:** Gene Ontology biological process categories enriched by genes differentially expressed in DM relative to controls.

Gene Ontology ID	Biological process
Enrichment score: 8.38	
GO:0051082	Unfolded protein binding
GO:0006457	Protein folding

Enrichment score: 4.08	
GO:0006412	Translation

Enrichment score: 3.95	
GO:0015031	Protein transport
GO:0045184	Establishment of protein localization
GO:0008104	Protein localization
GO:0006886	Intracellular protein transport
GO:0034613	Cellular protein localization
GO:0070727	Cellular macromolecule localization
GO:0046907	Intracellular transport

Enrichment score: 3.6	
GO:0045454	Cell redox homeostasis
GO:0019725	Cellular homeostasis
GO:0042592	Homeostatic process

Enrichment score: 2.7	
GO:0006270	DNA replication initiation
GO:0006261	DNA-dependent DNA replication
GO:0006260	DNA replication
GO:0006259	DNA metabolic process
