# Supplementary material for: Epigenetic Studies Point to DNA Replication/Repair Genes as a Basis for the Heritable Nature of Long Term Complications in Diabetes
Source: J Diabetes Res. 2016 Feb 14;2016:2860780. doi: 10.1155/2016/2860780 (PMC4769771; doi:10.1155/2016/2860780)
Supplement: Supplementary file 1 — Supplemental Table 1: The supplemental table 1 data provides the names of the 51 genes that were identified as being over-expressed in the functional category DNA replication/DNA metabolism process group. A selection criteria of at least 2-fold differential expression in the DM condition relative to controls with a FDR cut-off of 0.05 as used. The table lists for each column, the ENTREZ ID #, the Gene Symbol, and the Gene Name. Some genes are unknown (indicated by zgc). [file 2860780.f1.pdf]

Supplemental Table 1. Genes of the *DNA Replication/DNA Metabolism* Functional Group.

ENTREZ ID #	Gene Symbol	Gene Name
406730	apex1	APEX nuclease (multifunctional DNA repair enzyme) 1
30430	dnmt1	DNA (cytosine-5-)-methyltransferase 1
317744	dnmt4	DNA (cytosine-5-)-methyltransferase 4
321084	dnmt7	DNA (cytosine-5-)-methyltransferase 7
373130	dffb	DNA fragmentation factor, beta polypeptide (caspase-activated DNase)
192338	mcm2	MCM2 minichromosome maintenance deficient 2, mitotin (S. cerevisiae)
192323	mcm3	MCM3 minichromosome maintenance deficient 3 (S. cerevisiae)
337598	mcm4	MCM4 minichromosome maintenance deficient 4, mitotin (S. cerevisiae)
192333	mcm7	MCM7 minichromosome maintenance deficient 7 (S. cerevisiae)
317679	ruvbl1	RuvB-like 1 (E. coli)
317678	ruvbl2	RuvB-like 2 (E. coli)
282615	smarca5	SWI/SNF related, matrix associated, actin dependent regulator of chromatin, subfamily a, member 5; similar to SWI/SNF related, matrix associated, actin dependent regulator of chromatin, subfamily a, member 5
326746	cbx1a	chromobox homolog 1a (HP1 beta homolog Drosophila)
83780	cry2b	cryptochrome 2b
83774	cry3	cryptochrome 3
83776	cry5	cryptochrome 5
402986	cry-dash	cryptochrome DASH
445395	esco2	establishment of cohesion 1 homolog 2 (S. cerevisiae)
322599	eif1axb	eukaryotic translation initiation factor 1A, X-linked, b
386707	fen1	flap structure-specific endonuclease 1
751713	gtf2h5	general transcription factor IIH, polypeptide 5 heat shock cognate
30671	hsp70	heat shock cognate 70-kd protein, like; MCM5 minichromosome maintenance deficient 5 (S. cerevisiae); heat shock cognate 70-kd protein; zgc:174006; similar to heat shock protein 8
406845	msh2	mutS homolog 2 (E. coli)
260437	msh6	mutS homolog 6 (E. coli)
556665	nfia	nuclear factor I/A
334315	orc3l	origin recognition complex, subunit 3-like (yeast)
324256	orc6l	origin recognition complex, subunit 6 homolog-like (yeast)
322059	pola2	polymerase (DNA directed), alpha 2
30440	prim1	primase polypeptide 1; hypothetical protein LOC100149101
30678	pcna	proliferating cell nuclear antigen
393475	prmt7	protein arginine N-methyltransferase 7
406435	rfc4	replication factor C (activator 1) 4
327491	rpa1	replication protein A1
65226	rpa2	replication protein A2
321726	rbb4	retinoblastoma binding protein 4
322129	rbb4l	retinoblastoma binding protein 4, like
393195	rnaseh2a	ribonuclease H2, subunit A
30740	rrm1	ribonucleotide reductase M1 polypeptide
30733	?	ribonucleotide reductase M2 polypeptide
558344	?	si:ch211-51m24.3
556995	35b8.5	si:dkeyp-35b8.5
553496	top1l	topoisomerase (DNA) I, like
569877	top2b	topoisomerase (DNA) II beta
393313	ube2nl	ubiquitin-conjugating enzyme E2N-like
445317	zgc:101000	zgc:101000
794796	zgc:101072	zgc:101072
494100	zgc:101644	zgc:101644
678521	zgc:136878	zgc:136878
393196	zgc:56310	zgc:56310
406546	zgc:77429	zgc:77429
406462	zgc:85772	zgc:85772
